# Incoherent Optical Frequency-Domain Reflectometry Based on Homodyne Electro-Optic Downconversion for Fiber-Optic Sensor Interrogation

**DOI:** 10.3390/s19092075

**Published:** 2019-05-04

**Authors:** Juan Clement, Haroldo Maestre, Germán Torregrosa, Carlos R. Fernández-Pousa

**Affiliations:** Engineering Research Center (I3E), Department of Communications Engineering, Universidad Miguel Hernández de Elche, Av. Universidad s/n, 03202 Elche, Spain; hmaestre@umh.es (H.M.); gtorregrosa@umh.es (G.T.); c.pousa@umh.es (G.R.F.-P.)

**Keywords:** reflectometry, incoherent optical frequency-domain reflectometry, frequency-shifted interferometry, fiber Bragg grating, Rayleigh backscattering, optical fiber sensing

## Abstract

Fiber-optics sensors using interrogation based on incoherent optical frequency-domain reflectometry (I-OFDR) offer benefits such as the high stability of interference in the radio-frequency (RF) domain and the high SNR due to narrowband RF detection. One of the main impairments of the technique, however, is the necessity of high-frequency detectors and vector network analyzers (VNA) in systems requiring high resolution. In this paper, we report on two C-band implementations of an I-OFDR architecture based on homodyne electro-optic downconversion enabling detection without VNA and using only low-bandwidth, high-sensitivity receivers, therefore alleviating the requirements of conventional I-OFDR approaches. The systems are based on a pair of modulators that are synchronized to perform modulation and homodyne downconversion at a reference frequency of 25.5 kHz. In the first system, we attain centimeter resolution with a sensitivity down to −90 dB using the modulation frequency range comprised between 3.2 and 14.2 GHz. In the second, we measured, for the first time using this approach, Rayleigh backscattering traces in standard single mode fiber with resolution of 6 m and a sensitivity of −83 dB by use of the 10.1–30.1 MHz range. These results show the feasibility of these simple, homodyne downconversion I-OFDR systems as compact interrogators for distributed or quasi-distributed optical fiber sensors.

## 1. Introduction

Incoherent optical frequency-domain reflectometry (I-OFDR) was devised soon after the inception of optical time-domain reflectometry (OTDR) as an alternative technique for the characterization of fiber lines [1,2,3,4] and optical devices [5,6]. I-OFDR systems are based on the determination of the baseband radio-frequency (RF) transfer function of the system under test by use of a vector network analyzer (VNA), which is subsequently transformed to the time domain in order to provide a reflectometric trace equivalent to that of conventional OTDR [7]. Technically, the method is an application of the standard time-domain analysis of circuits and transmission lines in the microwave band [8,9], but where the modulation tones are carried by optical waves and where the system of interest is the optical circuit connecting the optical modulator and the detector.

The potentiality of I-OFDR systems for both distributed and quasi-distributed fiber-optics sensing has been widely recognized due to the inherent stability of the RF interference in the optical incoherent domain, and also to the high SNR that may be attained by electrical filtering. This way, I-OFDR has been applied to the measurement of both narrowband or broadband, discrete or distributed reflectors, either in time or directly in the RF domain, including fiber Bragg gratings (FBG) [10,11]; interferometers [12,13,14]; rings [15,16], Rayleigh [17], Brillouin [18], and Raman scattering [19]; and composite systems FBG/Raman [20]. It has also been enriched with concepts from Microwave Photonics [10,21], and much effort has been dedicated to the incorporation of additional functionalities to the technique. Wavelength-resolved reflectograms have been obtained by sweeping the wavelength of a narrowband optical probe wave [11] or filtering the backreflected light under broadband illumination [22,23], and the addition of dispersive elements has been used to map Bragg wavelength shifts into RF time delays directly measurable by the VNA [24,25,26]. In addition, specific techniques for discrete narrowband reflectors, typically FBG, have been adapted to I-OFDR systems, such as slope-assisted dual-wavelength [27] or dual-filter [28] interrogation, or fast techniques relying on model-based search [29] or equally-spaced arrays of reflectors [30]. In a different context, I-OFDR techniques have also proven its viability under a different set of stringent requirements, as embedded systems occupying a single RF channel for in-line monitoring of sub-carrier multiplexed fiber lines using Rayleigh backscattering (RBS) [31,32].

Despite this wealth of applications, I-OFDR shows certain general limitations of practical relevance. Being an optical incoherent technique, I-OFDR systems are usually limited by dynamic range [7]. Moreover, the standard implementation requires the use of a complex system such as the VNA. In addition, high resolution, down to the mm scale [5,6], can only be attained at the price of modulation and detection bandwidths exceeding several tens of GHz.

One of the proposed solutions to these issues that has recently received renewed attention is the use of optical downconversion to demodulate the reflected or backscattered wave, thus avoiding the VNA and allowing for the use of low-bandwidth, high sensitivity receivers without sacrificing bandwidth. In the first demonstration, Dolfi and Nazarathy [6] used two 26.5-GHz modulators to perform heterodyne optical downconversion of the stepped modulating tone to an intermediate frequency of 50 kHz, reaching a resolution of 4 mm that still constitutes the lowest attained value. The same concept is applied in frequency-shifted interferometry (FSI) [33], where a single acousto-optic modulator provides modulation in the forward path and homodyne downconversion to baseband in the reverse path. Due to the limited bandwidth of these devices, extension to GHz or multi-GHz bandwidths requires the alternative use of electro-optic amplitude or phase modulators. At moderate bandwidths (<GHz), homodyne downconversion of the reflected light can be performed by operating the modulator only in the forward direction [23,24,33]. In multi-GHz systems, however, modulation is only efficient if the RF modulation tone travels through the electrode in the same direction as light, and so it requires the use of specific driving stages to operate the electrode bidirectionally [34,35], the change of the modulator’s design [36], or the use of two different modulators as in the original heterodyne experiment [6]. Being homodyne, however, the retrieved signal from each modulating frequency is a dc voltage level, and so these systems suffer from the low-frequency noise present in the front-end amplifier, noise that may eventually limit the system’s sensitivity.

In this paper, we present an amplitude-modulated (AM) I-OFDR system based on homodyne electro-optic downconversion that overcomes this last problem by introducing a low-frequency modulation of the modulation index in the downconverting path, following an approach similar to that of Schlemmer in conventional I-OFDR [4]. The reflectometer is based on commercially available components, and employs two different electro-optic modulators for modulation and downconversion, stages that can be, in principle, integrated into a single device using the same techniques as in [35]. The system is conceived as a versatile platform that permits the practical exploration of two fiber-optics sensor scenarios, namely the interrogation of FBG arrays and the capture of RBS, both in the C band. In the first case, we demonstrate sensitivities down to −90 dB with centimeter resolution in fiber at an input optical power of +10 dBm, surpassing the values achieved by FSI and lock-in detection at a much lower resolution [37]. In the second case, we show, for the first time to the best of our knowledge, RBS traces using I-OFDR with electro-optical downconversion, reaching −83 dB sensitivity and a resolution in the meter range at an input optical power of +8 dBm.

This paper is organized as follows. In Section 2 we present, with a certain level of detail, the theoretical background describing both the standard stepped I-OFDR technique based on VNA and the optical downcoversion procedure used here, together with a brief analysis of the calibration procedures. Section 3 describes the experimental system and the general signal processing techniques used to retrieve the reflectogram. Section 4 is devoted to the presentation and discussion of the results, and we end in Section 5 with our conclusions.

## 2. Theoretical Background

### 2.1. Amplitude-Modulated Incoherent Frequency-Domain Reflectometry

**Figure 1:**
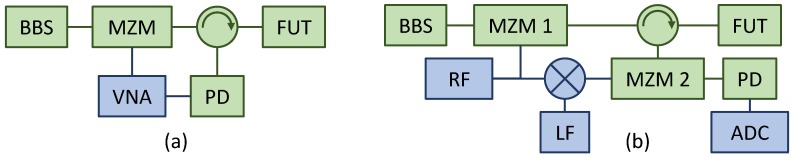
Schemes of stepped-frequency I-OFDR systems based on (**a**) vector network analysis; and (**b**) electro-optic downconversion. Photonic components are represented in green; RF and electronic components in blue.

I-OFDR systems are based on a modulated optical signal, used as a probe, whose modulating frequency is stepped over a certain bandwidth [1,2,3,4,5,6,7]. Whereas phase-modulated signals require specific demodulation schemes [35], AM waves can be directly detected by use of photodiodes. Due to its incoherent character, AM I-OFDR systems can be analyzed from power considerations as follows. Referring to Figure 1a, light from a broadband source (BBS) is modulated in amplitude by a Mach-Zehnder modulator (MZM). The power of this probe signal is given by

(1) $$P_{in}\left(t\right)=P_{0}T\left(1+\mu \cos(2\pi ft)\right)$$

where $P_{0}$ is the source’s optical power; *T* represents the modulator’s total loss, including the 3-dB loss due to quadrature bias; μ is the modulation index; and *f* is the RF modulating tone, extracted from the output port of the VNA. The probe signal (Equation (1)) is directed to a fiber under test (FUT), and the backreflected intensity converted to the electrical domain by use of a photodiode (PD). The received power is:

(2) $$P_{out}\left(t\right)=P_{0}T\;\left[R_{L}+\mu \left|H\left(f\right)\right|\cos\left(2\pi ft-\varphi (f)\right)\right]$$

where $R_{L}$ represents the return loss of the system under test, and the amplitude and phase variations of the modulated power are described by |H(f)| and −φ(f), respectively. These functions, which constitute the system’s RF transfer function in reflection, are retrieved by the VNA in complex form, H(f)=|H(f)|exp(−jφ(f)), after electronic mixing with the modulation tone of frequency *f*. Function H(f), if properly calibrated, can be presented in reflectivity units, as has been implicitly assumed in Equation (2). The reflectometric trace R(z) is retrieved by performing the inverse Fourier transform (IFT) of H(f), which yields the RF impulse response h(t), followed by the transformation from round-trip time to distance t=2z/v with *v* the fiber’s group velocity, $v=c/n_{g}$ with *c* the speed of light in vacuum and $n_{g}$ the group index, so that R(z)=|h(2z/v)|.

The impulse response depends on the bandwidth where H(f) is determined. In bandpass I-OFDR, the transfer function is retrieved between two non-zero frequencies, $f_{min}$ and $f_{max}$, with RF bandwidth $B=f_{max}-f_{min}$. In lowpass I-OFDR, $f_{min}=0$ and H(f) can be extended to negative frequencies by use of the hermiticity condition, $H\left(-f\right)=H{\left(f\right)}^{*}$, whereas the dc value is extracted by interpolation. This effectively doubles the IFT computation bandwidth, which is therefore ηB with η=1 for bandpass and η=2 for lowpass I-OFDR. Although all our experiments are bandpass I-OFDR, we maintain η in our discussion for the sake of generality.

In either case, the computation of the IFT is described by use of a window function W(f) that accounts for the bandwidth limitation, and also includes the possible windowing used to increase the measurement’s dynamic range [5,38]. The windowed impulse response is:

(3) $$h^{w}\left(t\right)=\int_{-\infty }^{+\infty }dfW\left(f\right)H\left(f\right)e^{j2\pi ft}=w\left(t\right)*h\left(t\right)$$

where w(t) is the IFT of the window W(f) and the asterisk denotes convolution. In a typical implementation, the RF transfer function is determined at a discrete number of *N* frequencies, $f_{k}=f_{min}+k\Delta f$, with k=1,…,N and Δf the scanning step, and the IFT leading to Equation (4) is performed as an inverse discrete Fourier transform (IDFT). In lowpass I-OFDR, the number of frequency steps is doubled to k=−N,…,N−1. The range of $h^{w}\left(t\right)$ is $t_{max}=1/\Delta f$, whereas the one-point resolution, or range bin size, expresses the separation between trace points and is given in terms of the bandwidth *B* as $\Delta t_{1}=1/\eta B$. The one-point resolution is a measure of the system’s capacity to locate the reflectivity’s peak value of a discrete reflector, and can be decreased by use of standard signal processing techniques, such as zero padding or the chirp Z-transform [38]. In turn, the two-point resolution, or range resolution, $\Delta t_{2}$, is the full width at half maximum (FWHM) of a peak in $|h^{w}\left(t\right)|$, and represents the minimum separation between reflectors that can be resolved by the instrument [7]. The range resolution can also be expressed in distance as $\Delta z=v\Delta t_{2}$, which is the physical distance illuminated by a virtual pulse of duration $\Delta t_{2}$ at a given instant. Under this point of view, we can map I-OFDR to OTDR parameters, and in fact we also use the OTDR displayed resolution, $W=v\Delta t_{2}/2$, which is the physical distance that contributes to the backscattering captured at a given instant. The factor 1/2 in W with respect to Δz accounts for the round-trip path of backscattering [39].

Let us now assume that the fiber line contains *M* point reflectors. The *k*th reflector, with reflectivity $R_{k}$, is located at distance $z_{k}$ with corresponding round-trip delay $\tau_{k}=2z_{k}/v$. The returned power signal is composed of a series of *N* delayed replicas of the AM signal with transfer function $H_{d}\left(f\right)=\sum_{k=1}^{M}R_{k}\;e^{-j2\pi f\tau_{k}}$, and the windowed impulse response is

(4) $$h_{d}^{w}\left(t\right)=\sum_{k=1}^{M}R_{k}\;w\left(t-\tau_{k}\right)$$

The two-point resolution is equivalent to the pulse width in a standard OTDR measurement since w(t) in Equation (3) or Equation (4) plays the role of a virtual OTDR input pulse. For a rectangular window, w(t)=sinc(ηBt), so that its FWHM is given by $\Delta t_{2}=1.21/\eta B$. This is the lowest attainable value, but shows a low sidelobe suppression of −6.6 dB. For a general window, $\Delta t_{2}=\kappa /\eta B$ with factor κ≥1.21 given by the window type. In our implementations, we include as the default window a Kaiser–Bessel window [38,40] with β=13, for which κ=2.8 and the sidelobe suppression ratio is −49 dB. In any case, the compromise between range and resolution is established through the number of frequency steps, since $t_{max}/\Delta t_{2}=\eta N/\kappa$.

The reflectogram associated to Equation (4) thus shows a series of *M* peaks at delays $\tau_{k}$ with heights $R_{k}w\left(0\right)$. For this reason, the window is normalized as w(0)=∫W(f)df=1, so that the peak readouts correspond to reflectivities. In addition, the determination of $H_{d}\left(f\right)$ in a practical, high-bandwidth system requires the subtraction of the non-optical RF response of cables, modulator, and photodiode. This can be accomplished by taking a “through” trace $H_{n}\left(f\right)$ with the modulator directly connected to the detector, and normalizing the experimental trace $H_{\exp}\left(f\right)$ with respect to this reference, so that $H_{d}\left(f\right)=H_{\exp}\left(f\right)/H_{n}\left(f\right)$ [5]. If the normalization is taken instead against a reflector of known reflectivity, such as the −14.4 dB Fresnel refection of a flat fiber end, the reflectogram is not only normalized but also calibrated. This has been the procedure followed in the present investigation. This procedure also leads to the determination of the sensitivity as the absolute noise level of the observed noise floor, computed as a rms or as a 98% value [39]. Finally, the dynamic range is defined as the difference between the highest measurable peak, typically that used for calibration, and the observed noise level in this configuration. This definition, which differs with respect to OTDR considerations and will be referred to as the *I-OFDR dynamic range*, constitutes a measure of the usable optical power range for the computation of the IDFT. Due to the incoherent character of the technique, typical values are limited to about 40–50 dB, and therefore halved with respect to the corresponding values in coherent systems [7].

**Figure 2:**
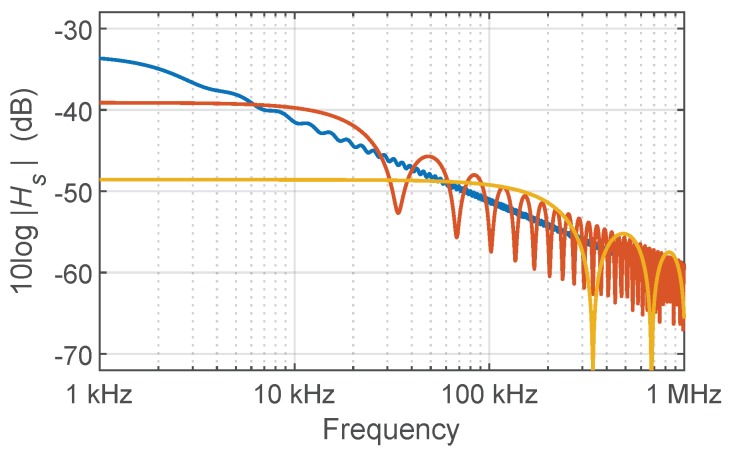
Backscattering transfer function $10\log\;|H_{s}\left(f\right)|$ of SMF at 1.55 μm for different fiber lengths: 30 km (blue), 3 km (orange), and 300 m (yellow).

Compared to discrete reflections, the RF transfer function of RBS shows a different structure. It is given by [1,2,3]:

(5) $$H_{s}\left(f\right)=\frac{S\alpha_{s}}{2\alpha }\frac{1}{1+jf/f_{a}}\left(1-e^{-2\alpha L}e^{-j2\pi f(2L/v)}\right)$$

with *S* the backscatter capture ratio, $f_{a}=\alpha v/2\pi$ the attenuation cut-off frequency, $\alpha_{s}$ the Rayleigh attenuation coefficient, and α the total attenuation coefficient. The IFT of Equation (5) is an exponentially decaying impulse response restricted to the time interval 0≤t≤2L/v. Standard values for the parameters in Equation (5) in the case of single-mode fiber (SMF) at 1.55 μm are $S=1.2\times 10^{-3}$, $\alpha_{s}=3.9\times 10^{-5}$ Np/m, $\alpha =4.6\times 10^{-5}$ Np/m, and $n_{g}=1.47$ [7], so that $f_{a}=1.5$ kHz. At low frequencies $H_{s}\left(f\right)$ tends to the total return loss, and above $f_{a}$ shows an overall amplitude decay of 10 dB per decade. This effect is known as the *high frequency issue* of RBS in I-OFDR [31,32], and was believed in the early stages of I-OFDR development to prevent its measurement [41]. This overall decaying trend is modulated with oscillations whose amplitude increases and whose spectral period decreases as *L* decreases, as exemplified in Figure 2.

To avoid the high frequency issue, our RBS measurements have been limited to a maximum frequency of 30.1 MHz and to frequency spans of 20 MHz, for which normalization was not necessary. As for the calibration, the readout level at t→0 is given by:

(6) $$h_{s}^{w}\left(0\right)=w\left(t\right)*h_{s}{\left(t\right)|}_{t=0}≃\frac{S\alpha_{s}v}{2}\int_{-\infty }^{\infty }w\left(t\right)dt$$

where we have neglected attenuation and assumed that the virtual pulse w(t) is entirely contained within the initial section of the fiber. In practice, the value of the right-hand side of Equation (6) is obtained by extrapolating the $|h_{s}^{w}\left(t\right)|$ trace to zero distance. Compatibility of different measurements thus requires that windows be normalized as W(0)=∫w(t)dt= constant [42]. This means that all windows applied to the same bandwidth *B* are chosen so that w(t) carry the same energy in the time domain, although they can present different resolutions.

The calibration of the RBS trace is not performed through a reference reflector, but after the identification with a particular virtual probe pulse. In detail, the initial readout level $|h_{s}^{w}\left(0\right)|$ is set by convention to the standard RBS coefficient σ [39] of a virtual square pulse whose width is the FWHM of the sinc pulse associated to the rectangular window [42]:

(7) $$|h_{s}^{w}\left(0\right)|\equiv \sigma =10\log\left(S\alpha_{s}W\right)=10\log\left(S\alpha_{s}\frac{v\Delta t_{2}}{2}\right)$$

with $\Delta t_{2}=1.21/\eta B$. With this definition, the initial RBS level of traces with double bandwidth differ by 3 dB and, in turn, traces with the same bandwidth but with different windows, and therefore with different values of κ, show the same initial RBS level, but differ in resolution. In our measurements, σ is the standard RBS coefficient of Corning’s SMF28 fiber at 1.55 μm, −82 dB at a pulse width of 1 ns. Finally, presentation is provided in 10log scale. This scale implies that RBS dynamic range, now defined, as usual, as the difference between the initial RBS level and the noise level, differs by a factor of two with the standard OTDR scale. Since in I-OFDR the reflective events located at the fiber’s input must be well resolved to perform the IFT, part of the 40–50 dB I-OFDR dynamic range in 10log scale is sacrificed, so that OTDR-like dynamic ranges, in 5log scale, are lowered to about 10 dB [31,32].

### 2.2. I-OFDR Using Homodyne Electro-Optic Downconversion

Referring now to Figure 1b, let us consider that instead of direct detection and VNA analysis we synchronously remodulate the received power $P_{out}\left(t\right)$ (Equation (2)) at the same stepped frequency *f* by use of a second modulator (MZM 2) driven by a RF signal generator (RF in Figure 1b) in order to downconvert to baseband the modulation tone. We temporally discard the mixer in Figure 1b. The remodulated power is:

(8) $$\begin{array}{cc} P_{out}^{\prime }\left(t\right)= & P_{out}\left(t\right)\times T^{\prime }\left[1+\mu^{\prime }\cos\left(2\pi ft\right)\right]\\ = & P_{0}TT^{\prime }\;\left[R_{L}+\mu \left|H\left(f\right)\right|\cos\left(2\pi ft-\varphi (f)\right)\right]\times \left[1+\mu^{\prime }\cos\left(2\pi ft\right)\right] \end{array}$$

with $\mu^{\prime }$ and $T^{\prime }$ the modulation index and loss of the second modulator, respectively. Let us assume that the detector’s bandwidth is lower than any modulating tone *f*. The detector’s dc output represents therefore the homodyne signal $X_{f}$ associated to the stepped frequency *f*, given by:

(9) $$\begin{array}{c} X_{f}=P_{out}^{\prime }{\left(t\right)|}_{dc}=P_{0}TT^{\prime }\left[R_{L}+\frac{1}{2}\mu \mu^{\prime }\left|H\left(f\right)\right|\cos\left(\varphi (f)\right)\right] \end{array}$$

Here, the desired information is the in-phase component of the RF transfer function, $|H\left(f\right)|\cos\left(\varphi (f)\right)$. As shown in Figure 1b, this component is retrieved by passing the modulating tone *f* through a double balanced mixer, and so the waveform driving the second modulator is $\mu_{0}^{\prime }\cos\left(2\pi f_{0}t\right)\cos\left(2\pi ft\right)$, with the *reference tone* $f_{0}$ extracted from a low-frequency signal generator (LF) and lying typically in the kHz range, within the lowest-noise detection band. This is equivalent to a low-frequency modulation of the modulation index, $\mu^{\prime }=\mu_{0}^{\prime }\cos\left(2\pi f_{0}t\right)$ in Equation (9). The homodyne signal $X_{f}$, now sinusoidally dependent in time, is digitized by an analog-to-digital converter (ADC in Figure 1b) at a sampling rate $f_{s}\geq 2f_{0}$, and the in-phase component of the RF transfer function extracted by digital filtering at the reference frequency $f_{0}$ followed by synchronous demodulation, as is described in the following section. The return loss term in Equation (9) is thus filtered and detection isolated from the low-frequency receiver’s noise. Comparison with Equation (2) indicates that the output incurs in an additional loss of $T^{\prime }\mu_{0}^{\prime }/4$ with respect to the level $P_{0}T\mu$ of a standard I-OFDR system based on VNA. This loss includes the downconversion loss $T^{\prime }\mu_{0}^{\prime }/2$ in Equation (9) and the additional 3-dB demodulation loss at $f_{0}$. For typical values $T^{\prime }=5$ dB and $\mu_{0}^{\prime }=0.5$, this represents an additional power penalty of 14 dB, which should be recovered by the increase in sensitivity provided by low-noise, amplified low-frequency receivers.

In the simplest case of a series of discrete reflectors, the in-phase component of the RF transfer function can be understood as a *spectral interferogram*, since it is composed of a sum of *M* oscillations of the form $R_{k}\cos\left(2\pi f\tau_{k}\right)$ with spectral periods $1/\tau_{k}$ determined by the reflector’s position and with amplitudes proportional to their reflectivities. Each of these terms can be interpreted as the result of the interference between the modulation sidebands after its return from the reflector, and the modulation sidebands locally generated from the carrier in the second modulation. This is the standard point of view in FSI systems [33].

In general, the electro-optic modulators used in the implementation of Equation (9) are operated at relatively high modulation indices to minimize modulation and downconversion losses, and therefore both modulation stages typically show significant harmonic distortion. In conventional stepped frequency I-OFDR with only the first modulation stage, distortion is not detrimental since higher-order harmonics are filtered by the VNA’s RF receiver. In turn, harmonic distortion when two modulators are used results in observable echoes of reflective events at double or triple distances, associated, respectively, to second- and third-order distortion. The echo at double distance is thus absent when the modulators are biased at quadrature. The detailed analysis of distortion is out of the scope of the present investigation.

Finally, polarization effects constitute another general limitation of the technique, since the probe optical signal can only be effectively modulated and remodulated in a linear polarization state if conventional electro-optic Mach–Zehnder modulators are used. Polarization mode dispersion and environmental effects such as local stress, orientation, and temperature changes within the fiber, together with the intrinsic polarization dependence of reflectors, may cause different responses to different input polarizations. Polarization control at the receiver’s side mitigates this problem, but this option is only viable for low ranges. The standard approaches to overcome this issue include the use of polarization-maintaining fiber [43]; the use of depolarizers or polarization scramblers to average the response over polarization at the price of an additional 3 dB power penalty [23]; or the use of polarization switching [43,44]. In our measurements of point reflectors, we implemented polarization switching, as described below. As for the RBS measurements, we adopted the point of view that the second modulator defines a polarization axis at the receiver, and thus the system is similar to a polarization OTDR, but based on I-OFDR techniques.

## 3. Experimental System and Signal Processing

**Figure 3:**
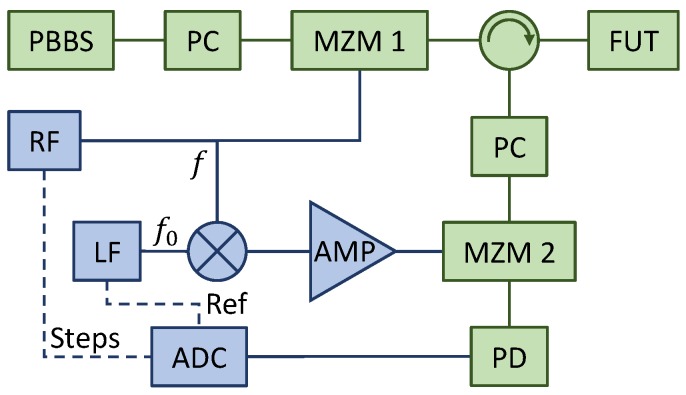
System setup. ADC, analog-to-digital converter; AMP, RF amplifier; LF, low-frequency signal generator; FUT, fiber under test; MZM, Mach–Zehnder modulator; PBBS, polarized broadband source; PC, polarization controller; PD, photodiode; RF, stepped RF source. Dashed lines stand for TTL signaling indicating RF steps (Steps) and reference tone (Ref).

The experimental setup is depicted in Figure 3. A 20-nm-wide polarized broadband source (PBBS) was modulated by a Mach–Zehnder modulator (MZM, JDSU APE AM-150) and launched into a FUT through a circulator. The reflected signal was modulated by a second MZM (Avanex PowerBit SD-20) and detected by a high-sensitivity, low-bandwidth amplified photodiode (Koheron PD01-AC-400). To maximize the modulation efficiency, a polarization controller was placed before each MZM to align the polarization to the modulator’s axes. The typical modulation indices of both modulators Were in the 0.5–0.7 range. Regarding the RF subsystem, the output of a stepped RF signal generator (Anritsu MG3692B) was split, driving the first MZM in one path and the mixer’s local oscillator port in the other. The system was configured in two different settings using commercial splitters and mixers, covering the 3.2–4.2 GHz range for the high resolution measurement of discrete reflections, and the 10.1–30.1 MHz band for low-resolution RBS measurements. The mixer’s intermediate frequency port was fed by the reference tone at $f_{0}=$ 25.5 kHz from a signal generator (TTi TG4001), and the resulting mixed signal drove the second MZM. To compensate for the mixer’s conversion losses, the signal was boosted by a RF amplifier adapted to the corresponding band. Finally, a digital board (Digilent Analog Discovery 2), working at a sampling frequency $f_{s}=$ 80 kHz and with a depth of 12 bits, digitized the photodiode’s output as well as recorded two TTL signals describing the initial and final instances of the time interval associated to each stepped frequency $f_{k}$ from the RF source, and the reference tone $f_{0}$ from the signal generator. These two signals are denoted as *Steps* and *Ref* in Figure 3, respectively.

A typical frequency sweep was composed of *N* different RF tones $f_{k}$ (k=1,…,N) which, after digitization, were described by an homodyne discrete-time signal $X_{f_{k}}\left[n\right]$ (Equation (9)) of length $L_{k}$ and oscillating at $f_{0}$. The signed amplitude of this oscillation, representing the in-phase component of the transfer function, was retrieved by first filtering the TTL reference signal at $f_{0}$ and then a Hilbert transform to obtain a reference tone in complex form, $\exp(j2\pi f_{0}n/f_{s}+j\theta )$, with $n=0,\ldots L_{k}-1$ and θ an arbitrary phase, which accounts for the different propagation delays between the *Ref* TTL signal and the reference tone passing through the mixer, amplifier, MZM and photodiode. Then, the complex amplitude at this frequency $f_{k}$ was computed by use of the IDFT:

(10) $$A_{c}\left[k\right]=\frac{1}{L_{k}}\sum_{n=0}^{L_{k}-1}\;X_{f_{k}}\left[n\right]\;\exp\left(j2\pi f_{0}n/f_{s}+j\theta \right).$$

**Figure 4:**
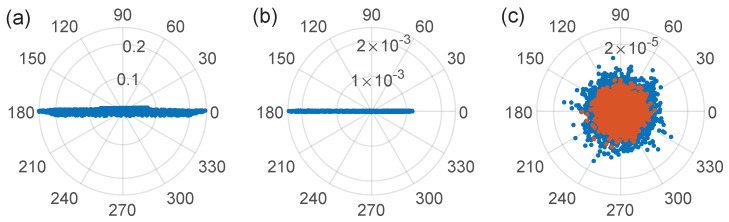
Interferogram constellations $A_{c}\left[k\right]\exp\left(-j\theta \right)$ after in-phase demodulation and rotation of: (**a**) a flat fiber end; (**b**) a 10-km SMF Rayleigh backscattering; and (**c**) system’s noise level when the RF signal is turned off but the optical source is on (blue), and when both RF and optical sources are off (orange), also for a 10-km SMF spool. The number of points in each constellation, or number of I-OFDR frequency steps $f_{k}$, is 5000.

Finally, to get the in-phase component or signed amplitude A[k] of the oscillation, the complex array $A_{c}\left[k\right]$ of length *N*, associated to the stepped frequencies $f_{k}$, was aligned to the real axis by use of principal component analysis:

(11) $$A\left[k\right]=ℜ\left\{A_{c}\left[k\right]\exp\left(-j\theta \right)\right\},$$

where $ℜ\left\{\cdot \right\}$ stands for real part and θ is estimated as the principal component angle of the constellation $A_{c}\left[k\right]$. Figure 4 shows representative constellations after rotation, associated, to (Figure 4a) a flat fiber end, (Figure 4b) RBS in 10 km of SMF Rayleigh backscattering, and (Figure 4c) measurements of the noise level when the reflectometer is operated without light (orange) and with light but without stepped frequencies (blue). We ascribed the noisier behavior of the flat fiber end to the wider frequency range, 10 GHz, needed to resolve a point reflection, what implies larger variations of the combined transfer function. In contrast, the backscattering measurement was performed between 10.1 MHz and 30.1 MHz, a narrower range where amplitude variations of the global transfer function are not perceptible. The asymmetry of the constellation in Figure 4b was due to an unbalanced distribution of the constellation points because of the wrapping of noisier samples around the zero-voltage level. Furthermore, Figure 4c shows the noise level, mostly dominated by the photodiode’s amplifier [31].

**Figure 5:**
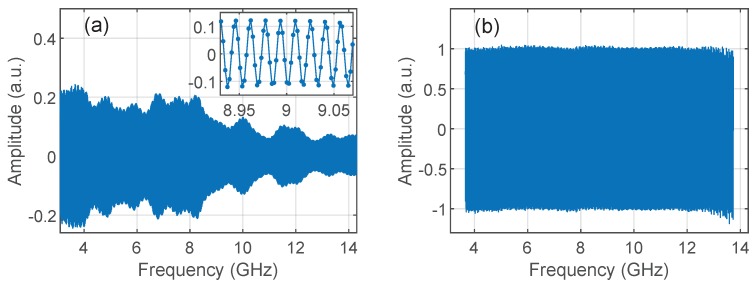
(**a**) Spectral interferogram $H_{n}\left[k\right]$ used for normalization and calibration, where the FUT is the reflection in a flat fiber end located 1 m after the circulator. Inset: Zoom over the 9-GHz region. Points are $H_{n}\left[k\right]$ samples; (**b**) Normalized and calibrated interferogram H[k], where the flat fiber end is situated 3 m after the calibration plane. Notice the constant level in a 10-GHz bandwidth and the trimming of H[k].

The second processing step was normalization. When dealing with wideband sweeps, the homodyne signal A[k] is prone to show large decays on its envelope due to variations of the amplitude of the global transfer function, as shown in Figure 5a, which results in a loss of resolution after the final IDFT. Since wideband, and therefore high-resolution, sweeps were targeted to discrete reflectors, the homodyne signal took the form of a spectral interferogram. To normalize the bandwidth, we retrieved the complex envelope $H_{\exp}\left[k\right]$ of the spectral interferogram A[k] by use of a Hilbert transform, and normalized it by the corresponding trace $H_{n}\left[k\right]$ of a flat fiber end. The final transfer function was therefore given by $H\left[k\right]=H_{\exp}\left[k\right]/H_{n}\left[k\right]$, from which the impulse response was computed by use of a window function and the final IDFT. The initial and final values of signal H[k] were slightly trimmed before IDFT to get rid of a transient behavior produced by the numerical Hilbert transform, as shown in Figure 5b, and therefore improving the time-domain response at the expense of a small resolution loss associated to an effective bandwidth of 10.08 GHz. The normalization procedure was not necessary with low-bandwidth sweeps, such as those used in RBS measurements.

## 4. Results

**Figure 6:**
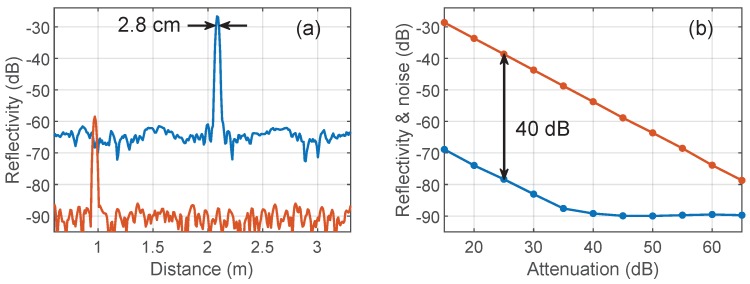
(**a**) System measurements limited by dynamic range (attenuated PC-air reflection, red) and sensitivity, (APC-air reflection, blue); and (**b**) peak (orange) and rms noise floor (blue) levels for progressively attenuated PC-air reflections.

In a first series of experiments, we characterized our bandpass I-OFDR system with broadband discrete reflectors in a total bandwidth of 10.08 GHz and an input optical power, at the output of the circulator, of +10 dBm. Traces were retrieved after maximizing the detected power after the second modulator by use of the polarization controller, and calibrated at a reflectivity of −24.4 dB using a flat fiber end and a 5-dB attenuator in double pass. We used a Kaiser–Bessel window with β = 13, thus the expected two-point resolution was 2.8 cm, a figure that can be lowered to 1.2 cm using a rectangular window. The systems under test were a couple of high and low reflective events, implemented through PC and APC connectors, the first after an addition of ∼6-dB of attenuation to keep the optical power below the maximum rating of the photodetector. The results, shown in Figure 6a, describe, respectively, a dynamic-range limited measurement and a sensitivity limited measurement. In the first, we observed the expected two-point resolution and a I-OFDR dynamic range of 40 dB; in the second, the absolute rms sensitivity reached −90 dB. The system’s measurement range could be estimated as ∼75 dB, measured from the sensitivity floor to the −15 dB reflectivity value associated to the maximum power handled by the photodiode. To further confirm the values of sensitivity and dynamic range, we performed and additional experiment where the PC-ended patchcord was preceded by a variable attenuator. Figure 6b shows the evolution of peak and noise levels for this configuration when the round-trip attenuation was progressively increased. The value of the dynamic range was similar to that obtained in the original, high-resolution, I-OFDR demonstrations [5,6] and also to the more recent baseband, 2-GHz conventional I-OFDR presented in [42]. The 98% sensitivity of this last reflectometer reached a value of −92 dB, but obtained at an input power of +13.5 dBm and after 3 min of acquisition time.

Regarding the trade-off between range and two-point resolution, our system was ultimately limited by the 10.08-GHz bandwidth and the maximum number of frequency steps allowed by our RF generator, $N_{\max}=10,000$. These values restricted the range to ∼45 m at the maximum resolution. However, in this first characterization, we only employed N= 5000 steps, as the associated >20 m range met the requirements of the tested FUTs. That also permitted to extend the dwell time of each frequency step up to 20 ms, ensuring a total acquisition time ≲2 min.

**Figure 7:**
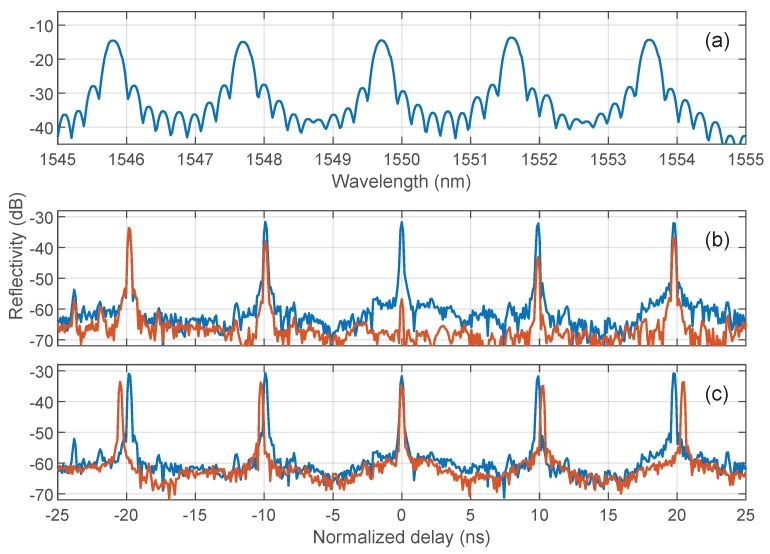
(**a**) FBG array measurement with orthogonally polarized light; (**b**) addition of traces on (**a**)) with (orange) and without (blue) CFBG right before down-converting MZM; and (**c**) FBG array reflectivity spectrum with resolution bandwidth 0.5 nm (blue) and 0.06 nm (orange).

In a second set of experiments, we explored a commercial C-band WDM array of five equally-spaced FBG (Draw Tower Grating from FBGS, inscribed in low bend loss fiber, LBL-1550-125) with length of 4 mm, reflectivity of 8.7±0.5%, FWHM of 0.18 nm, and a nominal separation between FBGs of 1 m. The Bragg wavelengths were mutually separated by 2 nm, as shown in Figure 7a. The observed reflectivity was lower due to the losses by mode mismatch between SMF and LBL fiber. This WDM array was chosen to perform a comparison of reflectivities measured with an OSA.

To take into account polarization changes in the paths between FBGs, we performed two different measurements using polarization switching between two orthogonal states of polarization. The second polarization controller was placed before the circulator, and then adjusted to the maximum and minimum reflectivity of the central FBG as displayed in an OSA placed after the second modulator. This assured that the light from this reflector reached the detector in the two orthogonal linear polarizations defined by the modulator’s axes, since it acted as a polarizer for the modulated wave. In addition, it was not difficult to show that these two configurations also assured that the light from the rest of reflectors also reached the detector in orthogonal polarizations, but not necessarily linear. The optical input power in this experiment was reduced to +4 dBm and calibrated by a flat fiber end without additional attenuation. Figure 7b shows the reflectograms in units of relative delay with respect to the central FBG. These two traces were finally summed, as depicted in Figure 7c in blue trace. The values of the reflectivity, calibrated with respect to a broadband reflector, were around −31 dB, approximately 15 dB lower than the reflectivities measured in the OSA due to the narrowband character of the FBGs. Relative reflectivity values between different FBGs were also compared with those of the OSA, showing a maximum deviation among the five FBGs as low as ±0.2 dB after taking into account the BBS spectral density at the different Bragg wavelengths.

In a final measurement, we included a dispersive element before the second modulator to illustrate the determination of dispersion-induced relative delays, a procedure which was used to measure relative Bragg wavelength shifts in FBG arrays [24,25,26]. The dispersive element was a chirped fiber Bragg grating (CFBG) with dispersion D=−170 ps/nm in the C band. The reflectogram is depicted in Figure 7b with an orange trace, showing a decrease in reflectivity level of 2.9 dB due to the CFBG insertion loss, and a shift in the FBGs peaks. Since the results are presented by normalizing the global delay of the central FBG, the relative shifts were about ±340 ps for the first pair of gratings, and ±680 for the second, being their mutual wavelength separation 2 nm. It can be also noticed how the peaks associated to the input connectors to the FBG array, located at ±23.7 ns in the blue trace, disappeared in the orange trace. This was because the temporal response of connectors, being wideband reflectors, was smeared in time due to the presence of the dispersive element.

**Figure 8:**
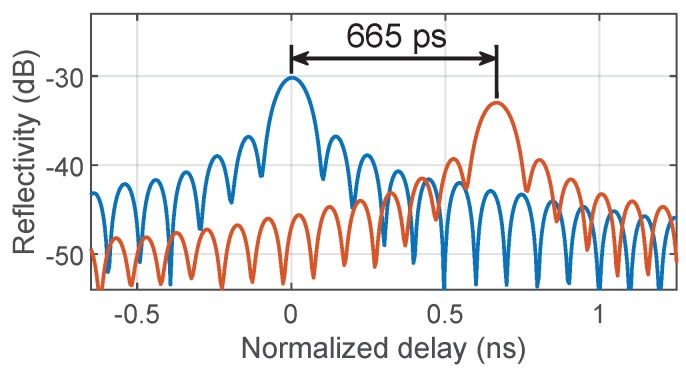
Zoom view of the two rightmost reflective peaks of Figure 7c, corresponding to the FBG at ∼1554 nm, with (blue) and without (orange) dispersive element. The window used here is rectangular.

A zoom view of the rightmost pair of peaks in Figure 7c, normalized in time with respect to the blue trace, is presented in Figure 8. In this example, we employed a rectangular window to ease the visualization of the peaks, and increased the one-point resolution of the plot by zero padding to reach the picosecond scale, which corresponded to a precision of 6 picometers in the determination of Bragg wavelength shifts at our value of dispersion. Again, this performance is similar to that obtained in conventional I-OFDR systems [25,26]. Moreover, the system’s architecture is, in principle, compatible with other forms of incorporating wavelength selectivity [11].

**Figure 9:**
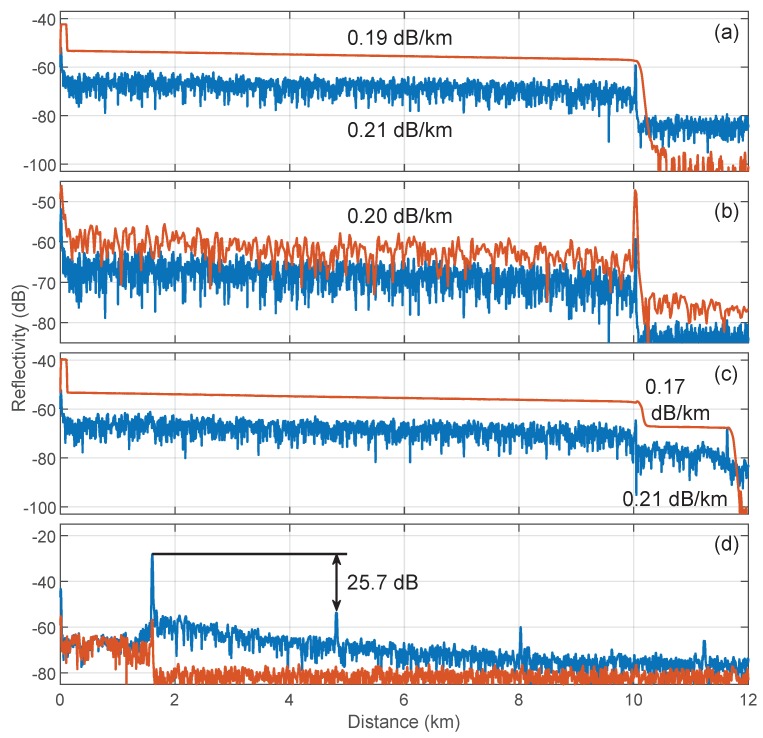
(**a**) Reflectometric traces, in 10log scale, of a 10-km spool of SMF using I-OFDR (blue) and a commercial OTDR (orange); (**b**) I-OFDR traces for 10-km SMF with B=20MHz (blue) and B=5MHz (orange); (**c**) I-OFDR (blue) and commercial OTDR (orange) measurements for the 10-km SMF coil followed by a 3-dB attenuator and 1.6 km of SMF; and (**d**) I-OFDR traces for 1.6-km SMF ended in PC (blue) and APC (orange) connectors. The acquisition time was 30 s at a pulse width of 1 μs for the OTDR and ≲2 min for the I-OFDR.

Finally, we performed a set of experiments targeted to the determination of RBS in several combinations of SMF spools using a mixer and a RF amplifier in a frequency band from $f_{min}=10.1$ MHz to $f_{max}=30.1$ MHz. The use of a lower frequency band, where the I-OFDR signal is higher, was not possible using our RF signal generator. First, the time-domain responses of a 10-km SMF coil ended in an APC connector are depicted in Figure 9a, measured with down-converted I-OFDR (blue) and with a standard OTDR at a 1-μs pulse width (orange). Both, as well as those in the following subplots, were calibrated following Equation (7), and showed a one-point resolution of 6 m. We observed in this subplot the wavy appearance of the I-OFDR trace, similar to that of polarization OTDRs [45]. We also depict the attenuation values obtained using both methods. In the I-OFDR trace, the fiber attenuation was determined by fitting the RBS in linear scale to an exponential decay, avoiding the fiber sections near the initial and final reflective events. The low discrepancies in the attenuation coefficient were ascribed to the random polarization of the received wave, which should be isolated from reflective events and averaged over a large number of spatial samples.

The observed rms sensitivity in Figure 9a was −83 dB, which differed with respect to the high-bandwidth configuration presented above due to the change in RF components and to the additional 3-dB loss due to the polarization-dependent detection. The dynamic range, measured from the initial RBS level to the noise floor, was 21 dB, which corresponded to 10.5 dB in 5log scale and represented a value similar to that obtained in [31] using the conventional I-OFDR approach.

In Figure 9b, we illustrate the effect of reducing the acquisition bandwidth, by limiting the maximum frequency to $f_{max}=15.1$ MHz (orange) and therefore reducing the one-point resolution by a factor of four and increasing the RBS level by 6 dB. The 20-MHz bandwidth trace, in blue, is also plotted for the sake of comparison. In Figure 9c, a 1.6-km SMF spool is added after the 10 km through a 3 dB optical attenuator to illustrate the detection of a local fault. The expected 6-dB round-trip decay with a dead zone limited by the two-point resolution, typical of I-OFDR measurements, is clearly visible at 10 km. Finally, as shown in Figure 9d, we measured the echoes originated by modulators’ harmonic distortion in a typical configuration. In this plot, the 1.6-km spool was measured standalone (orange) and with a 1-m APC-PC fiber section connected at the farther end through a 10-dB attenuator (blue) to simulate a highly reflective end. Echoes at odd multiples of this high-reflectivity end are visible, the third being the dominant 25.7 dB below the main peak. In the absence of highly reflective events, these echoes are harmless as they lie below the noise level, as shown in the orange trace.

Globally, the performance of this I-OFDR reflectometer for RBS characterization is similar to that of conventional I-OFDR systems based on VNA for sensor interrogation [17,42] or in-line monitoring [31], but with the identified limitations of polarization-dependent detection and echoes induced by harmonic distortion. The first can be solved by incorporating polarization switching [43,44] or scrambling [23]; the second represents a deterministic impairment that can be avoided, if necessary, by a previous calibration of the distortion and subsequent equalization.

## 5. Conclusions

In this paper, we report on an I-OFDR system based on electro-optic downconversion as a platform to explore the general capacities of this approach in fiber-optics sensor interrogation. The main advantages of this concept are the absence of dedicated high-frequency electronic components and the use of low-bandwidth, high-sensitivity receivers that overcome the power penalties associated to optical downconversion, thus enabling the implementation of competitive I-OFDR systems in terms of overall cost. On the downside, the amplitude-modulated system presented here shows echoes associated to modulator’s harmonic distortion, and requires specific polarization scrambling or switching stages to provide polarization-independent measurements.

In our setup, modulation and downconversion have been implemented by use of two different MZM, which can be integrated into a single device using bidirectional modulation. Moreover, the homodyne downconversion procedure based on modulation of the modulation index has permitted the use of a low-frequency band for detection, without the requirement of the more complex heterodyne downconversion with two swept tones separated by an intermediate frequency. The general performance of the reflectometer is, overall, similar to equivalent systems based on the conventional I-OFDR approach for fiber-optics sensor interrogation of point or distributed reflectors. In addition, our system has provided, for the first time to the best of our knowledge, measurements of RBS in SMF using downcoversion, measurements that can be improved in sensitivity by use of bands of lower frequency. In conclusion, the results show the feasibility of this simple approach for the implementation of compact, general-purpose I-OFDR interrogators for distributed or quasi-distributed optical fiber sensors.
